# Analgesic and Anti-Inflammatory Activity of *Pinus roxburghii* Sarg.

**DOI:** 10.1155/2012/245431

**Published:** 2012-06-14

**Authors:** Dhirender Kaushik, Ajay Kumar, Pawan Kaushik, A. C. Rana

**Affiliations:** ^1^Institute of Pharmaceutical Sciences, Kurukshetra University, Kurukshetra 136 119, India; ^2^Rayat College of Pharmacy, Ropar, Punjab 144 533, India

## Abstract

The Chir Pine, *Pinus roxburghii*, named after William Roxburgh, is a pine native to the Himalaya. *Pinus roxburghii* Sarg. (Pinaceae) is traditionally used for several medicinal purposes in India. As the oil of the plant is extensively used in number of herbal preparation for curing inflammatory disorders, the present study was undertaken to assess analgesic and anti-inflammatory activities of its bark extract. Dried and crushed leaves of *Pinus roxburghii* Sarg. were defatted with petroleum ether and then extracted with alcohol. The alcoholic extract at the doses of 100 mg/kg, 300 mg/kg, and 500 mg/kg body weight was subjected to evaluation of analgesic and anti-inflammatory activities in experimental animal models. Analgesic activity was evaluated by acetic acid-induced writhing and tail immersion tests in Swiss albino mice; acute and chronic anti-inflammatory activity was evaluated by carrageenan-induced paw oedema and cotton pellet granuloma in Wistar albino rats. Diclofenac sodium and indomethacin were employed as reference drugs for analgesic and anti-inflammatory studies, respectively. In the present study, the alcoholic bark extract of *Pinus roxburghii* Sarg. demonstrated significant analgesic and anti-inflammatory activities in the tested models.

## 1. Introduction

Inflammation is the response to injury of cells and body tissues through different factors such as infections, chemicals, and thermal and mechanical injuries [1]. Most of the anti-inflammatory drugs now available are potential inhibitors of cyclooxygenase (COX) pathway of arachidonic acid metabolism which produces prostaglandins. Prostaglandins are hyperalgesic, potent vasodilators and also contribute to erythema, edema, and pain. Hence, for treating inflammatory diseases, analgesic and anti-inflammatory agents are required [2]. Nonsteroidal anti-inflammatory drugs (NSAIDs) are the most clinically important medicine used for the treatment of inflammation-related diseases like arthritis, asthma, and cardiovascular disease [3]. Nonsteroidal anti-inflammatory drugs (NSAIDs) are among the most widely used medications due to their efficacy for a wide range of pain and inflammatory conditions [4]. However, the long-term administration of NSAID may induce gastro-intestinal ulcers, bleeding, and renal disorders due to their nonselective inhibition of both constitutive (COX-1) and inducible (COX-2) isoforms of the cyclooxygenases enzymes [5–7]. Therefore, new anti-inflammatory and analgesic drugs lacking those effects are being searched all over the world as alternatives to NSAIDs and opiates [8, 9]. Medicinal plants are believed to be an important source of new chemical substances with potential therapeutic effects. The research into plants with alleged folkloric use as pain relievers, anti-inflammatory agents, should therefore be viewed as a fruitful and logical research strategy in the search for new analgesic and anti-inflammatory drugs [10].

*Pinus roxburghii* Sarg. is the only tree with an ornamental specimen and having different medicinal values found in the Himalayan region of Bhutan, Nepal, Kashmir, Sikkim, Tibet and other parts of North India [11]. The plant is belonging to family Pinaceae commonly known as Chir Pine [12]. It consists of 110–120 species distributed throughout temperate regions of the Northern Hemisphere, and more than 40 taxonomic treatments have been recognized of several major divisions within the genus [13].

*Pinus roxburghii* Sarg. has many medicinal uses, the wood is aromatic, deodorant, haemostatic, stimulant, anthelmintic, digestive, liver tonic, diaphoretic, and diuretic. It is useful in eye, ear, and pharynx diseases, foul ulcers, haemorrhages, haemoptysis, worn infections, flatulence, liver diseases, bronchitis, inflammations, skin diseases, pruritus, and giddiness [11].

The chief chemical constituents of turpentine oil from *Pinus roxburghii* Sarg. are *α*-pinene, *β*-pinene, car-3-ene and longifolene [14, 15] hydrocarbons (d- and l-pinene), resin acids, camphene, fenchene, dipentene, and polymeric terpenes [16, 17]. 

Based on the above findings, *Pinus roxburghii *Sarg. bark extract was evaluated for its analgesic and anti-inflammatory effects on experimental induced pain and inflammation.

## 2. Material and Methods

### 2.1. Plant Material

The stems bark of* Pinus roxburghii* Sarg. were collected from the hilly region of Morni, District Panchkula, Haryana, in the month of December 2008 and was authenticated by FRI, Dehradun, Uttarakhand, India, where a voucher specimen no. 129 FHH was deposited for future reference.

### 2.2. Preparation of Extract

Shade dried coarse powdered bark of *Pinus roxburghii *Sarg. in a quantity sufficient as per the volume of extractor was packed in thimble (made of filter paper sheet). A sufficient volume of alcohol was added to the reservoir, and hot continuous extraction process in a Soxhlet extractor was started. This extraction process was continued for about 48 hours or until alcohol coming down the siphoning tube became colourless. The excess of alcohol was distilled under reduced pressure using rotatory vacuum evaporator. (Heidolph Laborota 4011, digital). A brown residue was recovered from flask with 12% yield.

### 2.3. HPLC Analysis

Samples of alcoholic bark extract of *Pinus roxburghii *Sarg. were analysed without any treatment.

The HPLC system (Shimadzu, Japan) consisted of a diode array detector (SPDM10AVP), solvent delivery module (LC-10ATVP), online degasser (DGU-14A), an autoinjector (SIL-10ADVP), flow channel system (FCV-14AH), system controller (SCL-10AVP), and a reversed-phase HPLC column (RP-18, 250 mm × 4.6 mm, 5 *μ*m particle size, Sigma, USA). The flow rate of the HPLC was 1 ML/min, and the mobile phase 0.05% TFA in ACN: 0.05% TFA in water (gradient) for 70 min. Standards of chlorogenic acid, rutin and querctin were injected separately (10 ML). Chemical compounds in the samples were identified by comparison of their retention times (Rt) with the standards. Data analysis was carried out using Class VP V6.12 SP2 software (Shimadzu, Japan).

### 2.4. Animals

Wistar rats (150–250 gm) and Swiss albino mice (20–25 gm) of either sex, brought from National Institute of Pharmaceutical Education and Research, Mohali, were kept in the Animal House of Institute of Pharmaceutical Sciences, Kurukshetra University, Kurukshetra. Animals were housed at standard conditions of temperature (22 ± 1°C) and 12/12 h light/dark cycle. They were fed with standard pellet diet (Ashirwad Industries, Ropar, Punjab) and had free access to water. Five animals are used in each group. Permission for conduct of these experiments were obtained from, Institutional Animal Ethics Committee (IAEC).

### 2.5. Acute Toxicity Study

Toxicity studies conducted as per internationally accepted protocol drawn under OECD guidelines 425 in Swiss albino mice.

## 3. Pharmacological Activity

### 3.1. Anti-Inflammatory Activity

#### 3.1.1. Carrageenan Induced Paw Edema Method

Carrageenan-induced paw inflammation was produced according to the method described by Winter et al. [18]. One hour after oral administration of the alcoholic extract of *Pinus roxburghii *Sarg. (100, 300, and 500 mg/kg), reference drug (indomethacin, 10 mg/kg) or vehicle (tween 80 (5%)), an injection of 0.1 ML of carrageenan (1% carrageenan suspended in 0.9% NaCl) was made into the right hind limb of each rat under the subplantar aponeurosis.

Measurement of paw volume was done by means of volume displacement technique using plethysmometer (Ugo Basile no. 7140) immediately after carrageenan injection and after 1, 2, 3, and 4 hr.

Percetages of inhibition were obtained using the following ratio:

(1)(Vt−Vo)  control−(Vt−Vo)  treated(Vt−Vo)  control×100.

*V*_*t*_ is the average volume for each group after treatment, and *V*_*o*_ is the average volume for each group before any treatment. 

#### 3.1.2. Cotton Pellet Granuloma Method

Cotton pellet granulomas produced according to the method described by Winter and Porter [19]. Sterile cotton pellets (20 ± 0.5 mg) were implanted subcutaneously in the abdomen region of the rats. The animals received alcoholic bark extract of *Pinus roxburghii *Sarg. (100, 300 and 500 mg/kg), reference drug (diclofenac sodium, 50 mg/kg) or vehicle (tween 80 (5%)), orally, once a day through an oral cannula over seven consecutive days. On the 8th day, the rats were sacrificed, the cotton pellets removed, pellets dried up to constant weight at 60°C and the net dry weight, that is, after subtracting the weight of the cotton pellets, was determined.

### 3.2. Analgesic Activity

#### 3.2.1. Acetic Acid Induced Writhing Test Method

The method used in this test has been described by Koster et al. [20]. The total number of writhings following intraperitoneal administration of acetic acid solution (1%, 10 mL/kg) was recorded over a period of 10 min, starting 5 min after acetic acid injection. The mice were treated with the alcoholic bark extract of *Pinus roxburghii *Sarg. (100, 300, and 500 mg/kg), or vehicle (tween 80 (5%)) or standard drug (diclofenac sodium, 50 mg/kg), 30 min before administration of acetic acid. The number of writhings and stretching was recorded and permitted to express the percentage of protection.

#### 3.2.2. Tail Immersion Test in Rats

The procedure described by Aydin et al. [21] was used to conduct this test. 3 cm of the tail was introduced in hot water at a temperature of 55 ± 0.5°C. Within a few minutes, the rats reacted by withdrawing the tail. The reaction time was recorded with a stopwatch. The animals were treated by alcoholic extract of *Pinus roxburghii *Sarg. (100, 300 and 500 mg/kg), or water (vehicle) or standard drug (diclofenac sodium, 50 mg/kg), 30 min before the immersion of the tail. The time reaction is taken at 1, 2, 3, and 4 after administration of different preparations.

#### 3.2.3. Statstical Analysis

All values were expressed as mean ± SEM, and data was analyzed by one way analysis of variance (ANOVA) followed by Dunnett's  *t*-test using GraphPad InStat.

## 4. Results 

### 4.1. Acute Toxicity Test of Plant Extract

Alcoholic extract of the plant *Pinus roxburghii* Sarg. was found safe at the dose of 5000 mg/kg according to OECD guidelines 425.

### 4.2. HPLC Analysis

**Figure 1:**
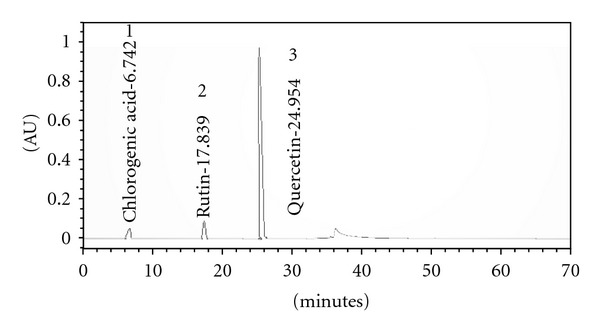
HPLC analysis of  *Pinus roxburghii* Sarg. (Pinaceae) showing presence of chlorogenic acid (1), rutin (2) and quercetin (3).

A correct assignment to the various compounds was not possible. From UV spectra and retention times of the main peaks, some compound classes contained in the extract have been determined. High-performance liquid chromatography (HPLC) revealed the presence of bioflavonoids, quercetin, chlorogenic acid, and rutin (Figure 1).

### 4.3. Anti-Inflammatory Activity

#### 4.3.1. Carrageenan-Induced Paw Edema in Rats

In the carrageenan-induced oedema test, the paw volumes and percentages of ages of inhibition by the alcoholic extract of *Pinus roxburghii* Sarg. and standard drugs are shown in Table 1. Injection of carrageenan was done 1 h after oral administration of the extract (100, 300, and 500 mg/kg), indomethacin (reference drug) and water. Measurement of paw size was taken before carrageenan injection and then 1, 2, 3, and 4 h after carrageenan injection. The alcoholic extract of *Pinus roxburghii* Sarg. at all doses showed a significant inhibition of paw oedema at third hour as compared to reference drug (Table 1).

#### 4.3.2. Cotton Pellet Granuloma

Table 2 shows that the alcoholic bark extract of *Pinus roxburghii* Sarg. exhibited a significant and dose-related inhibition of the dried weight of the cotton pellet granuloma. The inhibitory values for 100, 300, and 500 mg/kg of the extract were 31.11%, 36.13% and 56.93% (*P* > 0.01), respectively. Diclofenac sodium (reference drug) and water inhibited granuloma tissue formation with a value of 92.87 (*P* > 0.01) %, a slightly higher value than that observed with the 80 mg/kg dose of the *Pinus roxburghii* Sarg. extract.

### 4.4. Analgesic Activity

#### 4.4.1. Acetic Acid Induced Writhing Test in Mice

The alcoholic extract of *Pinus roxburghii* Sarg. (100, 300, and 500 mg/kg) dose significantly and dependently reduced the number of abdominal constriction induced in mice by a solution of acetic acid 1%. This dose-dependent protective effect reached a maximum inhibition of 80.95% at the dose of 500 mg/kg. Diclofenac sodium (reference drug) exerted a significant protective effect, with percentage of protection of 90 (Table 3).

#### 4.4.2. Tail Immersion Test in Rats

As presented in Table 4, alcoholic extract of *Pinus roxburghii* Sarg. in doses of 500 mg/kg (*P* < 0.01) body weight showed a significant elongation of reaction time, 30 minutes after oral administration of the extract. After 60 minutes, the alcoholic extract of *Pinus roxburghii* Sarg. in doses of 300 mg/kg (*P* < 0.05) and 500 mg/kg (*P* < 0.05) body weight showed a significant elongation. After 90 minutes alcoholic extract of *Pinus roxburghii* Sarg. in doses of 300 mg/kg (*P* < 0.05) and 500 mg/kg body weight showed a significant (*P* < 0.05) elongation of reaction time. After 120 minutes alcoholic extract of *Pinus roxburghii* Sarg. in doses of 100 mg, 300 mg, and 500 mg/kg body weight showed no significant elongation of reaction time.

## 5. Discussions

Carrageenan-induced edema has been commonly used as an experimental animal model for acute inflammation and is believed to be biphasic. The early phase (1-2 h) of the carrageenan model is mainly mediated by histamine, serotonin, and increased synthesis of prostaglandins in the damaged tissue surroundings. The late phase is sustained by prostaglandin release and mediated by bradykinin, leukotrienes, polymorphonuclear cells, and prostaglandins produced by tissue macrophages [10, 22]. Since the extract/fractions significantly inhibited paw edema induced by carrageenan in the second phase, this finding suggests a possible inhibition of cyclooxygenase synthesis by the extract and this effect is similar to that produced by nonsteroidal anti-inflammatory drugs such as indomethacin, whose mechanism of action is inhibition of the cyclooxygenase enzyme. 

The inflammatory granuloma is a typical feature of an established chronic inflammatory process [23, 24]. The cotton pellet granuloma method has been widely employed to evaluate the transudative, exudative, and proliferative components of chronic inflammation, because the dried weight of the pellets correlates well with the amount of granulomatous tissue [25]. We found a dose-dependent inhibition of granuloma formation in mice, suggesting that the aqueous stem bark extract of *Pinus roxburghii* Sarg. inhibits chronic inflammation processes during the late phases of acute inflammation.

The brain and spinal cord play a major role in central pain mechanisms. The dorsal horn of the spinal cord is endowed with several neurotransmitters and receptors including substance P, somatostatin, neuropeptide Y, inhibitory amino acid, nitric oxide, endogenous opioids, and the monoamines which are the major targets for pain and inflammation [26]. The tail immersion test was considered to be selective to examine compounds acting through opioid receptor; all the extract/fractions increased pain threshold which means basal latency, which indicates that it may act *via* centrally mediated analgesic mechanism. Narcotic analgesics inhibit both peripheral and central mechanism of pain, while nonsteroidal anti-inflammatory drugs inhibit only peripheral pain [27]. The extract inhibits pain with both mechanisms, suggesting that the plant extract may act as a narcotic analgesic. On the other hand, acetic acid-induced writhing model represents pain sensation by triggering localized inflammatory response. Such pain stimulus leads to the release of free arachidonic acid from the tissue phospholipid [28]. The acetic acid induced writhing response is a sensitive procedure to evaluate peripherally acting analgesics. The response is thought to be mediated by peritoneal mast cells [29], acid sensing ion channels [30], and the prostaglandin pathways [31]. Flavonoids may increase the amount of endogenous serotonin or may interact with 5-HT_2_A and 5-HT_3_ receptors which may be involved in the mechanism of central analgesic activity [32]. Moreover, EtOAc extract showed highest analgesic activity in all the experimental modelo which may be due to its high flavonsid contents which are responsible for free radical scavenging activity, as these free radicals are involved during pain stimulation, and antioxidants showed reduction in such pain [33].

The results of the present study have shown that the crude extract of the investigated plant exhibited very high anti-inflammatory and analgesic activities. These activities may be linked with the presence of polyphenolic compounds present in the extract. The HPLC analysis of AB extract shows the presence of bioflavonoids, quercetin, and rutin, which are reported to be anti-inflammatory, antiasthmatic, analgesic anti-inflammatory, and antioxidant, and these findings are in concordance with our results. Many plants containing flavonoids have been shown to have diuretic, laxative, antispasmodic, anti-hypertensive, and anti-inflammatory actions [34]. Flavonoids and saponins are well known for their ability to inhibit pain perception as well as anti-inflammatory properties due to their inhibitory effects on enzymes involved in the production of the chemical mediator of inflammation [35].

The ability of flavonoids to inhibit eicosanoid biosynthesis has been documented. Eicosanoids, such as prostaglandins, are involved in various immunological responses and are the end products of the cyclooxygenase and lipoxygenase pathways [36]. Further, flavonoids are able to inhibit neutrophils degranulation and thereby decrease the release of arachidonic acid [37]. Thus, the presence of flavonoids in the extract/fractions of *Pinus roxburghii* Sarg. might be responsible for the anti-inflammatory and analgesic activity in Swiss albino mice and rats.

## Figures and Tables

**Table 1:** Protective efeect of *Pinus roxburghii* Sarg. on paw edema induced by carrageenan in rat.

		Change in paw volume (mL) Mean ± SEM			
Groups	Drug (dose), route	Time (hr)			
		1 hr	2 hr	3 hr	4 hr
Control	Tween 80 (5%) p.o	0.71 ± 0.12	0.93 ± 0.01	1.8 ± 0.03	1.9 ± 0.02
Standard	Indomethacin (10 mg/kg), p.o	0.21 ± 0.06 (70.4)	0.23 ± 0.02** (75.26)	0.41 ± 0.01** (77.22)	0.35 ± 0.09 (81.56)
AB	100 mg/kg, p.o	0.61 ± 0.17 (14.08)	0.92 ± 0.17 (1.07)	0.89 ± 0.13** (50.55)	1.07 ± 0.19 (43.68)
AB	300 mg/kg, p.o	0.63 ± 0.14 (11.21)	0.78 ± 0.19 (16.12)	0.79 ± 0.20** (56.11)	1.87 ± 0.14 (1.57)
AB	500 mg/kg, p.o	0.39 ± 0.12 (45.07)	0.52 ± 0.13 (44.08)	0.68 ± 0.11** (62.22)	0.68 ± 0.11 (64.21)

*n* = 5. Results are expressed as mean ± SEM. Percentage inhibition are in brackets.

*P > 0.05, **P < 0.01 as compared to control, AB = alcoholic bark extract.

**Table 2:** Protective efeect of *Pinus roxburghii* Sarg. on cotton pellet induced granuloma.

Groups	Drug (dose), route	Weight of dry Cotton Pellet Granuloma (mg)	% protection
Control	Tween 80 (5%) p.o	202 ± 0.23	0
Standard	Indomethacin (10 mg/kg), p.o	14.4 ± 3.3**	92.87
AB	100 mg/kg, p.o	139 ± 3.2	31.11
AB	300 mg/kg, p.o	129 ± 1.1	36.13
AB	500 mg/kg, p.o	87 ± 9.1**	56.93

*n* = 5. Results are expressed as mean ± SEM. *P > 0.05, **P < 0.01 as compared to control, AB = alcoholic bark extract.

**Table 3:** Protective efeect of *Pinus roxburghii* Sarg. on writhing induced by acetic acid.

Groups	Drug (dose), route	No of wriths (Mean ± SEM)	% Protection
Control	Acetic acid (0.6% V/V), i.p	42 ± 11.6	0
Standard	Diclofenac Sodium (50 mg/kg), p.o	4.2 ± 1.0**	90
AB	100 mg/kg, p.o	25.4 ± 4.8	39.52
AB	300 mg/kg, p.o	17.4 ± 1.9**	58.57
AB	500 mg/kg, p.o	8.0 ± 2.0**	80.95

*n* = 5. Results are expressed as mean ± SEM. *P > 0.05, **P < 0.01 as compared to control, AB = alcoholic bark extract.

**Table 4:** Protective efeect of *Pinus roxburghii* Sarg. on tail withdrawal reflex induced by tail immersion.

Groups	Drug (dose), route	Reaction time (min) Mean ± SEM			
		30 min	60 min	90 min	120 min
Control	Tween 80 (5%), p.o	1.0 ± 0.1	1.4 ± 0.2	1.5 ± 0.2	2.8 ± 0.2
Standard	Diclofenac sodium (50 mg/kg), p.o	5.8 ± 0.2**	8.2 ± 0.2**	8.4 ± 0.2**	8.8 ± 0.2**
AB	100 mg/kg, p.o	1.4 ± 0.2	2.4 ± 0.3	2.8 ± 0.2	3.4 ± 0.2
AB	300 mg/kg, p.o	1.8 ± 0.2	3.2 ± 0.2*	4.4 ± 0.4*	6.2 ± 1.5*
AB	500 mg/kg, p.o	2.8 ± 0.4**	3.2 ± 0.4*	4.6 ± 0.2*	5.4 ± 1.9

*n* = 5. Results are expressed as mean ± SEM. *P > 0.05, **P < 0.01 as compared to control, AB = alcoholic bark extract.
